# Asymmetrical Polymer Vesicles for Drug delivery and Other Applications

**DOI:** 10.3389/fphar.2017.00374

**Published:** 2017-06-20

**Authors:** Yi Zhao, Xiaoming Li, Xiaotian Zhao, Yunqi Yang, Hui Li, Xinbo Zhou, Weien Yuan

**Affiliations:** ^1^School of Pharmacy, Shanghai Jiao Tong UniversityShanghai, China; ^2^School of Medicine, University of California, San FranciscoSan Francisco, CA, United States; ^3^Laboratory of Computer-Aided Drug Design and Discovery, Beijing Institute of Pharmacology and ToxicologyBeijing, China

**Keywords:** asymmetrical polymer vesicle, drug delivery, self-assemble, nano-delivery vesicles

## Abstract

Scientists have been attracted by polymersomes as versatile drug delivery systems since the last two decades. Polymersomes have the potential to be versatile drug delivery systems because of their tunable membrane formulations, stabilities *in vivo*, various physicochemical properties, controlled release mechanisms, targeting abilities, and capacities to encapsulate a wide range of drugs and other molecules. Asymmetrical polymersomes are nano- to micro-sized polymeric capsules with asymmetrical membranes, which means, they have different outer and inner coronas so that they can exhibit better endocytosis rate and endosomal escape ability than other polymeric systems with symmetrical membranes. Hence, asymmetrical polymersomes are highly promising as self-assembled nano-delivery systems in the future for *in vivo* therapeutics delivery and diagnostic imaging applications. In this review, we prepared a summary about recent research progresses of asymmetrical polymersomes in the following aspects: synthesis, preparation, applications in drug delivery and others.

## Introduction

Polymersomes, which also can be called polymeric vesicles, have attracted scientists' interests in recent years. Compared to other nanometer scale self-assembling delivery systems, such as polymer micelles, nanoparticles and nanogels, polymersomes provide a more stable condition for the storage of drugs, especially for nanoscopic emulsions (Asano et al., 2016). As compared to similar devices such as liposomes, which are comprised of low molecular weight lipids, polymersomes are composed of versatile macromolecular amphiphiles architectures, including dendritic copolymers (Yang et al., 2005), graft polymers (Dou et al., 2003; Lee et al., 2006), amphiphilic diblock polymers (Discher et al., 1999; Qin et al., 2006; Zhou et al., 2006), triblock polymers (Nardin et al., 2000; Napoli et al., 2004a) and so on. Because of this unique composition, polymersomes commonly exhibit much better colloidal stability, solidity, and protection of drugs, whereas illustrating very little chemical permeability. Composed of amphiphilic AB diblock or ABC triblock copolymers, polymersomes usually have relatively longer hydrophobic blocks to make up the dense core membranes, and shorter hydrophilic blocks in the facial membranes to protect the interior fluids in aqueous solutions. Hence, this advantage presents an excellent solution to the problem that no matter what hydrophilic or hydrophobic property a particular drug has, polymersomes can act as appropriate carriers for the drug. For example, both hydrophobic (e.g., doxorubicin, paclitaxel, quantum dots) and hydrophilic molecules (e.g., DNA, siRNA, therapeutic proteins, chelated Gd) can be encapsulated into polymersomes (Wang et al., 2012; Anajafi et al., 2017; Nomani et al., 2017).

As we all know, for a drug carrier, biodegradability is the first thing that must be considered carefully (Zhao et al., 2014). The alternative hydrophobic block-based drug carrier systems, such as liposomes, must be decorated with poly(ethylene glycol) (PEG) or polycarbonates in order to maintain a stealthy property. PEG is the most commonly used material for decorating drug carriers because it can maintain the carriers' stability in the plasma by prohibiting the adsorption of plasma proteins and has little toxicity to the cells (Kricheldorf, 2006). Hence, the carriers whose surfaces are decorated with PEG chains usually get longer blood circulation times (Gref et al., 2000; Ohya et al., 2011). However, this will not be a problem for polymersomes. Taking the advantage of the arbitrary structures, polymersomes can be intrinsically stealthy, because they present typical hydrophilic macromolecular amphiphilic parts (e.g., PEG and dextran) in the facial membranes. This non-fouling property offers scientists a great convenience to make further transformation. Though polymersomes have the above advantages over liposomes, polymersomes have relatively low loading levels and loading efficacies for hydrophilic drugs, including protein drugs and chemotherapeutics, which limits their applications as drug carriers, especially for anti-tumor therapy. To investigate this issue, great efforts have been made to improve the loading efficiency of polymersomes. It is a normal way to decorate polymersomes with specific ligands to create smart polymersomes, which selectively releases payloads to the sites of action in response to external stimulus (e.g., magnetic and photo) or internal signals (e.g., enzyme degradation, endo/lysosomal pH and cytoplasmic glutathione). Decorated materials usually include aptamers, lactoferrin, antibodies, peptides, and folate (Lu et al., 2015). Moreover, changing the permeability of the membranes, or controlling the degradability of the structures can also achieve the same effect (LoPresti et al., 2009). In addition, ingenious encapsulation procedures such as nano-precipitation method (Sanson et al., 2010) and transmembrane phosphate-, citrate-, sulfate-, or PH- gradient loading (Choucair et al., 2005; Ahmed et al., 2006a; Yin et al., 2009; Gubernator, 2011) are also smart ways to enhance the loading efficacy.

Lo Presti et al. have demonstrated that by exploiting endocytosis, pH-sensitive PMPC-PDPA polymersomes can deliver various drugs effectively to cells (Lomas et al., 2007, 2008; Massignani et al., 2009, 2010a; Murdoch et al., 2010). There exists different mechanisms that control the acidification of the internalized material within subcellular compartments (endosomes) (Doherty and McMahon, 2009). The authors have also found that after polymersomes are internalized by endocytosis, they will disassemble in a controllable manner when approaching the acidic endosomal lumen. This, conversely, leads to endosomal membrane perturbation in a short time, which will facilitate the polymersomes to escape from endosomes and get into the cell cytosol (Lomas et al., 2007, 2008; Massignani et al., 2009). The endocytosis efficiency is strongly dependent on the polymersome topology and surface chemistry, as well as the polymersome size (Massignani et al., 2009).

Although the difficulty can be conquered in various ways, it still remains a big challenge that symmetrical polymersomes exhibit inefficient intracellular drug delivery. First of all, as symmetrical polymersomes-based drug delivery systems show low endosomal escape ability, they may be trapped inside the endosomes. Besides, the side effects to healthy cells due to the slow endocytosis rate and drug diffusion can never be neglected. In recent years, asymmetric polymersomes have attracted a lot of attention in the drug delivery field. They have the following advantages: efficient drug loading capacity, fast endocytosis rate and endosomal escape ability, which can meet most requirements for drug delivery (Liu et al., 2014). Asymmetrical vesicles with different structures both inside and outside are self-assembled from asymmetric ABC triblock copolymers or AB and BC two diblock copolymers (Lu et al., 2015). In this review, we prepared a summary about recent progresses of asymmetrical polymersomes in the following aspects: synthesis, preparation, applications in drug delivery and others.

## Synthesis and preparation of asymmetrical polymersomes

### Synthesis of asymmetric triblock copolymers

There are two methods to synthesize the materials of polymersomes. One is mixing AB and BC diblock copolymers together, the other is directly using ABC triblocks to form asymmetrical polymersomes. Consisting of at least two homopolymer blocks, block copolymers are the ideal materials for forming self-assembling polymersomes. Normally, the homopolymer is designed to exhibit some specific physicochemical properties, and in consequence, the block copolymers will display versatile properties and utilization values (Lee and Feijen, 2012; Ge et al., 2014).

Sequential radical addition-fragmentation chain transfer (RAFT) polymerization is the most commonly used technique to synthesis triblock copolymers (Du et al., 2012). Ring-opening polymerization (ROP) is another feasible way to synthesize diblock or triblock copolymers (Kishimura et al., 2007). The properties of the diblock or triblock copolymers can determine the types and applications of the asymmetrical polymersomes by controlling the structures, compositions and molecular weights (Li et al., 2015). Therefore, we summarize some available diblock or triblock copolymers that constitute asymmetrical polymersomes, and the results are given in Table 1.

**Table 1:** Examples of available diblock or triblock copolymers that constitute asymmetrical polymersomes.

**Polymers**	**Preparation method**	**Formation method**	**Copolymers**	**Targeting ligands**	**Drugs/stimulus for release**	**Pros and cons**	**References**
PEO45-b-PS130-b-PDEA120	Atrp	None reported	PEO45-b-PS130-b-PDEA120	None reported	pH-induced	None reported	Giacomelli et al., 2007
FA/DTPA-PGA-b-PCL	None reported	Solvent switching method	FA-PGA75-b-PCL30 and DTPA-PGA22-b-PCL30	FA	DOX·HCl/pH-induced	Improve the sensitivity of a T2 MRI contrast agent	Oerlemans et al., 2010
Anis-PEG-PTTMA-PAA	Raft	Solvent switching method	Anis-PEG-PTTMA-PAA and PEG-PTTMA-PAA	Anisamide	GrB/pH-induced	Targeting ability and prompt intracellular protein release	Lu et al., 2015
PEG5K-P(CL-co-LA)11K-PEG2K	Rop	Film hydration method	mPEG-PCL or mPEG-P-(CL-co-LA)	None reported	Hb	Undamaged gas-binding capability and oxygen affinity, plus high stability and biocompatibility	Kishimura et al., 2007
PEO113-b-PCL132-b-PAA15	None reported	Solvent switching method	PEO113-b-PCL132-b-PAA15	None reported	DOX·HCl/pH-induced	High DOX loading efficiency and good biodegradability, rapid endocytosis rate and endosomal escape ability	Liu et al., 2014
Acupa-PEG-PTMBPEC-PSAC	Rop	Solvent switching method	Acupa-PEG-PTMBPEC-PSAC and PEG-PTMBPEC-PSAC	2-[3-[5-amino-2-carboxypentyl]-ureido]-pentanedioic acid	GrB/pH-induced	Unimodal distribution, high protein loading contents, long circulation time	Du et al., 2012
PEG-SS-PCL-PDEA	Rop	Solvent switching method	PEG-PCL-PDEA and PEG-SS-PCL	Galactose	GrB/reduction-induced	Unimodal distribution, highly efficient loading, high protein loading contents, long circulation time, target ability	Drummond et al., 1999
PB-b-PS	Atrp	Blending in an oil-in-oil emulsion	polystyrene-b-poly(ethylene oxide) (SO) and polybutadiene-b-poly-(ethylene oxide) (BO)	None reported	None reported	Straightforward preparation method	Asano et al., 2016
PEG-PTTMA-PAA	Raft	Solvent switching method	PEG-PTTMA-PAA	None reported	DOX·HCl/pH-induced	Conveniently prepared, high loading efficiency, excellent biocompatibility, quickly destabilized	Du et al., 2012
PEG–PAA(SH)–PDEA)	Raft	Solvent switching method	PEG–PAA(SH)–PDEA)	None reported	FITC–CC/reduction- and pH–induced	Conveniently prepared, high loading content, excellent biocompatibility	Zupancich et al., 2006
PEO-PAA-PNIPAM	Raft	Solvent switching method	PEO-PAA-PNIPAM	None reported	FITC–dextran/temperature-induced	Stability against high salt conditions and change of temperature	Du and O'Reilly, 2009
PEO–PDPA–PDMA	Atrp	Solvent switching method	PEO–PDPA–PDMA	None reported	None reported	None reported	Zhang and Eisenberg, 1995
PEG-PCL-PDEA	Raft	Film hydration method	PEG-PCL-PDEA	None reported	FITC-CC	High protein loading efficiencies and controlled release, able to simultaneously deliver and release hydrophobic anticancer drugs and proteins into cells	Liu et al., 2010
PEG-PCL-DEX	Rop	Solvent switching method	PEG-PCL and DEX-PCL	None reported	PEO	A variety of chemically dynamic characteristics responding to biological pathways	Zhang et al., 2010

### Preparation of asymmetrical polymersomes

There are two major methods to form self-assembling asymmetrical polymersomes: solvent change method (phase inversion) and direct hydration method (Zhang and Eisenberg, 1995; Blanazs et al., 2009). The first method is more widely used. Interestingly, Asano et al. first used the co-assembly method to generate asymmetric polymersomes. They put two distinct diblock copolymers PS-b-PEO (SO) and PB-b-PEO (BO) in an oil-in-oil emulsion PS/PB/CHCl_3_ (Asano et al., 2016).

## Drug delivery by asymmetric polymersomes

As mentioned above, compared with liposomes, polymersomes have more robust membranes, thus can improve the circulation half-life, protect drugs and prevent uncontrolled drug release. However, the rate and spatial distribution cannot be controlled because of the restriction of the structures (Mecke et al., 2006). Therefore, polymersomes that can response to various stimuli are developed by changing the physical and chemical properties of the membranes of the polymersomes (Du and O'Reilly, 2009; Onaca et al., 2009; van Dongen et al., 2009). As a consequence, the side effects are reduced and the efficacy of drugs at the site of action is improved. The stimuli can be classified into two aspects: external stimuli (temperature change, UV light and magnetic field) and intracellular stimuli (pH, redox).

### Temperature-responsive asymmetric polymersomes

Compared with UV stimuli for drug release, temperature stimuli are a more practicable way for building intelligent polymersomes, since natural temperature differences in tissues exist in human body. Tumor tissues have a higher temperature than normal tissues and the temperature can change easily in the external, such as hyperthermia (Xu et al., 2009). Furthermore, using the heating or cooling appliances for particular sections of the body can also achieve the effect of temperature differences (Christian et al., 2009).

**Figure 1:**
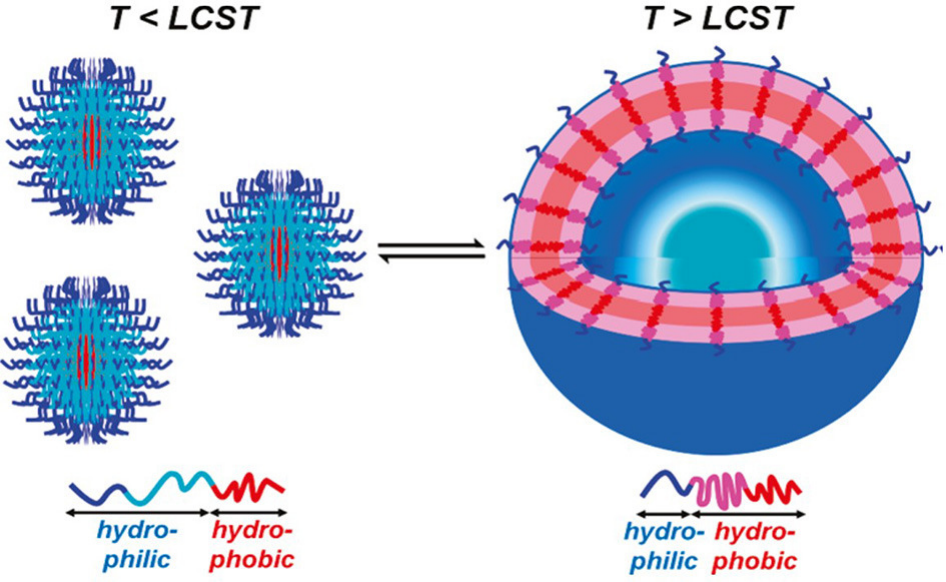
Schematic illustration of the thermally induced size change of assemblies of copolymers containing thermoresponsive blocks. Below the LCST, the central block (cyan) is hydrophilic; above the LCST, the central block (pink) becomes hydrophobic (Cai et al., 2011) (The figure has been obtained with copyright permission/license from ACS).

The temperature-dependent mechanisms can be achieved by using the polymer poly(N-isopropylacrylamide) (PNIPAm) (Li et al., 2006; Qin et al., 2006). PNIPAm has a unique property that its conformation will change if the temperature is above the lower critical solution temperature (LCST) of PNIPAm (40°C). Above 40°C, the block will change its property from hydrophilic to hydrophobic. Thus, temperature-sensitive polymersomes are designed by using PNIPAm. Usually, PNIPAm chains are covalently combined with a hydrophilic block such as poly(N-(3-aminopropyl) methylacrylamide hydrochloride) (PAMPA) or PEO. The resulting copolymer will self-assemble to form polymersomes and have the capacity to load hydrophilic drugs. If the temperature decreases below 40°C, the polymersomes will disassemble to the former copolymer and release the encapsulated therapeutics. Cai et al. (2011) prepared asymmetric polymersomes based on poly(ethylene oxide)-b-poly(ethylene oxide-stat-butylene oxide)-b-poly(isoprene) (E-BE-I) ABC triblock copolymer, which presented the temperature dependent property and the LCST was 25°C. Compared to the usual temperature-responsive polymersomes using PNIPAm as responsive factors, these polymersomes had narrower molecular weight distribution and faster transformation rate because of the lacking of strong interchain hydrogen bonding (Figure 1).

### pH-responsive asymmetric polymersomes

As a matter of fact, in the tumor microenvironment there is an innate pH difference (pH 6.5−7.2) and the cancer cells' endosomes and lysosomes are acidic (pH 4.0−6.5) (Grabe and Oster, 2001; Rofstad et al., 2006; Meng et al., 2014). Ahmed et al. found that a number of polymersomes approached cellular endolysosomes by pinocytosis passageway (Ahmed et al., 2006b). Hence, this intracellular cue can be applied for pH-dependent asymmetric polymersomes. This release method has an advantage over external cues (temperature, UV) owing to the accessibility of such intracellular cues. Using polymersomes whose structures are susceptibility to this pH difference, targeted intracellular release of the specific encapsulated cargo can be achieved. Du et al. investigated endosomal pH-sensitive degradable asymmetric polymersomes constructed by ABC triblock poly(ethylene glycol)-b-poly(trimethoxybenzylidene tris (hydroxymethyl)ethane methacrylate)-b-poly(acrylic acid) (PEG-PTTMA-PAA). In this study, they demonstrated that PTTMA block could quickly destabilize due to the degradation of acetal groups in lower pH in the endo/lysosomal compartments of cancer cells, achieving targeted intracellular release of DOX·HCl and high anti-tumor therapeutic effects (Du et al., 2012).

### Redox-responsive asymmetric polymersomes

Oxidation (Napoli et al., 2004b,a) or reduction-responsive (Cerritelli et al., 2007) reactions open up new spatial release mechanisms in the human body (Bodor, 1987; Corti et al., 2010). Oxidative environments exist at sites of inflammation, tumor tissues and in extracellular fluids, as well as intracellular compartments such as the endolysosomes (Grundl, 1994; Tew, 2007).

Wang et al. (2013) demonstrated reduction-responsive asymmetrical polymersomes based on PEG-SS-PCL diblock copolymer and asymmetric PEG-PCL-PDEA triblock copolymer. When exposed to reducing environments such as the nuclei and cytoplasm of cancer cells, this system will quickly rupture due to cleavage of the disulfide bonds between PEG and PCL blocks. This delivery system can protect encapsulated cargos in the extracellular environments (i.e., blood plasma) and induce efficient intracellular drug release, therefore it has the potential to be a versatile and multifunctional drug delivery platform by using intracellular stimuli (Krack et al., 2008).

## Applications of asymmetric polymersomes

Polymersomes have higher stability than liposomes (Discher et al., 1999; Photos et al., 2003; Lee et al., 2011). Moreover, polymersomes not only can encapsulate hydrophilic drugs but also can hold hydrophobic or amphiphilic compounds into their lumina or membranes. This advantage can be used in the fields of drug delivery, medical imaging and disease diagnosis (Massignani et al., 2010b). It has been proved that anticancer drugs (i.e., PTX and DOX·HCl) (Ahmed et al., 2006a; Chen et al., 2010), membrane proteins (i.e., OmpF, LamB, and FhuA, Stoenescu et al., 2004), hydrophobic dyes (i.e., PKH26) (Photos et al., 2003) and amphiphilic dyes (i.e., octadecyl rhodamine B, Battaglia and Ryan, 2005; Lomas et al., 2008) can be incorporated into membranes of polymersomes.

### Delivery of conventional drugs

Encapsulating drugs in asymmetric polymersomes can achieve efficient drug loading capacity, fast endocytosis rate and rapid endosomal escape ability. All of these advantages help to maintain the property of drugs *in vivo* or *in vitro* and control the release rate. With the development of smart delivery systems, many cancer-target chemotherapeutics drugs which have severe side effects now come to a renascence (Arrigo, 2000, 2005; Arrigo and Ducasse, 2002).

Liu et al. reported pH-sensitive asymmetric polymersomes based on PEO-b-PCL-b-PAA triblock copolymer. PEO chains constituted the outer corona of polymersomes because it showed well-behaved biocompatibility and was intrinsically stealthy to immune system. The pH-responsive PAA chains were designed in inner aqueous core since they could quickly destabilize at endosomal pH. Consequently, the release of encapsulated cargos in the cells was efficient and accurate (Liu et al., 2014).

### Delivery of protein

Protein drugs have been recognized as one kind of the most potential leads for the growth of new therapeutics (Tan et al., 2010), and they are powerful antidotes toward many intractable diseases such as diabetes and cancers (Torchilin and Lukyanov, 2003; Futaki, 2006; Schrama et al., 2006). However, they are too sensitive to environmental conditions, which results in their short lives *in vivo*.

Polymersomes with huge aqueous compartments can encapsulate proteins and the membranes will protect proteins from degradation (Discher et al., 2007; Christian et al., 2009; Sun et al., 2009; Meng and Zhong, 2011; Lee and Feijen, 2012; Liu et al., 2012). So far, polymersomes have encapsulated a variety of proteins, including aquaporin Z (Kumar et al., 2007), cytochrome C (CC) (Hvasanov et al., 2011), hemoglobin (Rameez et al., 2008; Li et al., 2012), insulin (Xiong et al., 2007; Christian et al., 2009; Kim H. et al., 2012), immunoglobulin G (Fu et al., 2011), ovalbumin (Stano et al., 2013), and myoglobin (Kishimura et al., 2007). Recent investigations also have found that polymer vesicles with asymmetrical membranes exhibit much higher loading capacity of proteins than symmetrical polymersomes.

Li et al. reported self-aggregated vesicles with asymmetric membranes. These polymersomes can serve as hemoglobin (Hb)-based oxygen carriers with oxygen affinity and high Hb loading content and meanwhile no interference with blood cells. Further studies have shown that these vesicles have great stability and efficacy, which have the potential to be alternative blood substitutes (Li et al., 2014).

### Theranostic vesicles for magnetic resonance imaging

Magnetic resonance imaging (MRI) is a widespread diagnosis apparatus that is clinically used for taking pictures of organs and structures inside the body to find blood vessels, bleeding, tumors or infection. Using asymmetric polymersomes to deliver contrast agents has better sensitivity and lower toxicity. Liu et al. (2015) reported asymmetrical polymersomes based on two kinds of diblock copolymer FA-PGA75-b-PCL30 and DTPA-PGA22-b-PCL30, which had diagnostic and therapeutic effects simultaneously. The highest drug DLE can reach 52.6% and the T1 relaxivity can be increased by 8-fold, meanwhile maintaining a low toxicity and remarkable positive contrast enhancement for tumor imaging.

### Applications of non-spherical shape asymmetric polymersomes

Polymersomes with non-spherical shapes play important roles in applications such as vaccine development. Non-spherical shape can cause different interaction to immune cells because the shapes determine the *in vivo* behavior of polymersomes. Actually, there are not comprehensive studies that investigated the influence of shape and topology on the *in vivo* behavior of polymersomes.

Researchers have found that tubular structures are one of the most promising morphologies rather than spheres. Owing to the resembling with bacterial topologies, tubular structures offer larger contact areas between particles and cells. Van Hest's group has developed a series of methods that reshaped spherical vesicles to tubular structures. The ability of tunable membrane gives these spherical structures a chance to apply to the immunology field (Williams et al., 2017). As for polymersomes, not only the copolymer composition can influence the morphology but also the response to various stimuli such as temperature, pH, magnetic fields, or osmotic pressure can further change the structures. In a recent example by van Hest et al. (Abdelmohsen et al., 2016a), the formation of functional nanotubes from PEG-PDLLA was demonstrated. Biodegradable polymersomes made by PEG-PDLLA copolymer transformed their structures from spheres to nanotubes upon dialysis under hypertonic conditions. Owing to the dialysis with the increasing concentration of NaCl, the size can be osmotically controlled by the enrichment and elongation of the structures. In this way, such well-defined nanoparticles can be easily prepared and functionalized. This method established a novel platform for biomedical research where nanoscopic control over size and shape is highly valuable. Another example of non-spherical vesicles is the stomatocytes, which can also be prepared by dialysis. Through shape transformation of spherical polymersomes into bowl-shaped stomatocytes, polymersomes will form an extra nanocavity. Due to the direct contact with the outside environment, these vesicles present the ability to convert chemical energy into kinetic energy when encapsulating enzymes or catalytic nanoparticles in the nanocavity. Further investigations showed that stomatocytes have the chemotactic ability toward certain cell types (Williams et al., 2017). Stomatocytes are promising candidates for further useful applications such as immunoassays, protein and DNA isolation, detection and biosensing (Abdelmohsen et al., 2016b).

### Applications of targeting polymersomes

Much efforts have been made for targeting drugs and genes to specific sites. It is crucial for clinical therapeutics because drugs without targeting ability may cause low effect and high toxicity. Targeted delivery is categorized into two types: active targeting and passive targeting. Passive targeting aims at capturing nanoparticles that are smaller than the aperture gap of endothelial cells. These nanoparticles can go through the interstitium and therefore gather in tumor tissue (Danhier et al., 2009). Active targeting takes advantage of molecular recognition to transport drugs to specific sites. Attaching biological ligands or antibodies to the nanoparticles is the most widely applied method and it is usually carried out by chemically conjugating (Yang et al., 2002; Li et al., 2005; Rerat et al., 2010), mixing (Guo et al., 2009) and coating (Yang et al., 2002; Elloumi Hannachi et al., 2009). Targeting moieties are usually conjugated to the hydroxyl groups of their hydrophilic polymer blocks (e.g., PEO, PEG) (Torchilin et al., 2001; Velonia et al., 2002; Levine et al., 2008). In addition, polymersomes conjugated with targeting moieties may change their hydrophilic-block-to-total-mass ratio, resulting in the change of morphology. For example, from vesicles to micelles.

Lu et al. reported the modification of their triblock copolymer (PEG-PTTMA-PAA) polymersomes with anisamide (Anis) to target the cancer cell receptor sigma (Lu et al., 2015). Sigma receptor is an over-expressed membrane protein that appears in many human malignant diseases including lung cancer and prostate cancer (Lu et al., 2015). Anis ligands exhibit high affinity to sigma receptor and so far have been used as guides to deliver versatile drugs including proteins, siRNAs and doxorubicin (Della Rocca et al., 2011; Guo et al., 2012; Kim S. K. et al., 2012). Their results validated that Anis-chimaeric polymersomes (CPs) presented high targeting ability to H460 and when Anis contents increased, antitumor efficacy was improved. If competitive antagonist is used, the antitumor activity will be reduced drastically (Lu et al., 2015).

### Applications of leukopolymersomes

Many polymersomes are easily internalized by endocytosis of macrophages and induce inflammatory responses. Therefore, polymersomes are often designed to be biodegradable and biocompatible and with reduced *in vivo* inflammatory responses.

Leukopolymersomes are a kind of polymersomes that have adhesive properties to leukocytes. It can be easily made by just functionalizing the terminal groups on the membranes of the vesicles. There are two typically adhesive ligands on leukocytes, which are named as selectins and integrin. These two ligands can mimic the adhesive properties of activated leukocytes (Hammer et al., 2008). Engineered to express the adhesion molecules, leukopolymersomes can achieve binding to inflammatory substrates in the shear fluid flow in blood vessels (Hammer et al., 2008). Moreover, by altering the membrane materials and ligand ratio, the rate and type of adhesive interaction can be tuned. Hammer et al. demonstrated that the adhesion was specific because the adhesive rate was the same as that of leukocytes (Hammer et al., 2008).

The adhesiveness of leukopolymersomes can be coupled with other properties of polymersomes, such as their ability to encapsulate drugs and image contrast agents. These particles will ultimately be useful for creating theranostic particles that can detect, image and deliver drugs to inflammatory sites, cancer cells, and cardiovascular lesions (Robbins et al., 2010).

## Conclusions

In this review, we summed up the recent progresses of asymmetrical polymersomes from the aspects of preparation, delivery, applications and targeting of asymmetrical polymersomes. Asymmetrical polymersomes with asymmetrical membranes and large watery cores can encapsulate various therapeutic molecules including both hydrophilic and hydrophobic molecules. Their tunable membrane formulations, stability *in vivo*, various physicochemical properties, controlled release mechanisms, and targeting ability make asymmetrical polymersomes one of the most ideal platforms for drug delivery. According to many recent papers, we have found that a large number of examples showing polymersomes research has caught up with liposomal science in the past decade and in many cases, adding extra dimensions to what is possible. In the future, more research should be performed to further improve the following aspects of asymmetrical polymersomes: (1) increasing the loading capacity and encapsulation rate of drugs into asymmetrical polymersomes; (2) enhancing the efficiency of controlled release with the goal of achieving zero-order release and stimuli-responsive release; (3) improving the delivery efficiency of macromolecular therapeutics such as protein and nucleic acid drugs; (4) improving the *in vivo* circulation half-life and the efficiency for specifically targeted drug delivery.

## Author contributions

YZ, XL, XZ, YY, HL, XZ, and WY: conceived and participated in its design, searched databases, extracted and assessed studies and helped to draft the manuscript. WY: conceived the initial idea and the conceptualization, participated in the data extraction and analysis, and revised the manuscript. XZ and HL: participated in the conceptualization and design of the study and analysis, and YZ: wrote the manuscript. All authors read and approved the final manuscript.

### Conflict of interest statement

The authors declare that the research was conducted in the absence of any commercial or financial relationships that could be construed as a potential conflict of interest.
